# Dedicated emergency department physical therapy is associated with reduced imaging, opioid administration, and length of stay: A prospective observational study

**DOI:** 10.1371/journal.pone.0231476

**Published:** 2020-04-23

**Authors:** Andrew Pugh, Keith Roper, Jake Magel, Julie Fritz, Nazaret Colon, Sadie Robinson, Caitlynn Cooper, John Peterson, Asal Kareem, Troy Madsen

**Affiliations:** University of Utah School of Medicine, Salt Lake City, Utah, United States of America; East Carolina University Brody School of Medicine, UNITED STATES

## Abstract

**Background:**

Emergency department based Physical Therapy (ED-PT) has been practiced globally in various forms for over 20 years and is an emerging resource in the US. While there is a growing body of evidence suggesting that ED-PT has a positive effect on a number of clinical and operational outcomes in patients presenting with musculoskeletal (MSK) pain, there are few published narratives that quantify this in the US. Although there are international papers that offer outcome data on reduction of pain, imaging, throughput time, and the ability of physical therapists to appropriately manage MSK conditions in the ED setting, most papers to date have been descriptive in nature. The purpose of this study is to assess the impact of ED-PT on imaging studies obtained, rates of opioids prescribed, and ED length of stay.

**Methods:**

We prospectively identified patients presenting with musculoskeletal pain to an urban academic ED in Salt Lake City between January 2017 and June 2018. During the study, a physical therapist was in the ED three days (24 hours) per week and was available to evaluate and treat patients after consultation by the ED provider. We noted patient demographic information, imaging performed in the ED, medications administered and prescribed, and ED length of stay. We classified patients as those who received PT in the ED and those who did not and compared clinical outcomes between groups. We performed a subgroup analysis of patients presenting with low back pain and matched patients by age and gender.

**Results:**

Over the 18-month study period, we identified 524 patients presenting to the ED with musculoskeletal pain. 381 (72.7%) received ED-initiated PT. The PT and non-PT groups were similar in average age (42.8 years vs. 45.1 years, p = 0.155), gender (% female: 53% vs. 46.9%, p-0.209), and primary presenting chief complaint (cervical, thoracic, or lumbar pain: 57.7% vs. 53.1%, p = 0.345). Patients who received PT had lower rates of imaging (38.3% vs. 51%, p = 0.009), ED opioid administration (17.5% vs. 32.9%, p<0.001), and a shorter average ED length of stay (4 hours vs. 6.2 hours, p<0.001). Rates of outpatient opioid prescriptions were similar between groups (16% vs. 21.7%, p = 0.129). In a subgroup analysis of patients presenting with low back pain, we found that PT patients had fewer imaging studies (PT 25% vs. non-PT 57%, p = 0.029) but found no difference in average ED length of stay (PT 3.7 hours vs. non-PT 4.6 hours, p = 0.21), opioid administration in the ED (PT 36% vs. non-PT 43%, P = 0.792), nor outpatient opioid administration (PT 17.9%. vs non-PT 17.9%, p = 1.0).

**Conclusion:**

In our experience, being seen by a physical therapist for MSK pain within the ED was associated with reduced use of imaging and time spent in the ED. Patients seeing a Physical Therapist were also less likely to receive an opioid prescription within the ED, a potentially significant finding given the need for opioid reduction strategies.

## Background

Musculoskeletal pain is a leading cause of disability globally and a common complaint in patients presenting to the emergency department (ED). [1,2] It is estimated that 13.8% of ED visits are attributable to primary musculoskeletal disorders. [3] Back pain, the most common of these disorders, accounts for 4.3 million ED visits per annum in the US. [2,4] When faced with managing non-traumatic musculoskeletal pain in the ED, physicians often have limited treatment options available. ED providers may find themselves trapped between conflicting mandates to alleviate pain and maintain patient satisfaction while practicing in accordance with national guidelines that recommend reduced opioid prescription and imaging. [5–8] Patients presenting to the ED with musculoskeletal pain may also have culturally conditioned expectations, such as an expectation to receive imaging to assist in their diagnosis and medication to ameliorate their symptoms. [7]

While the busy ED environment is not conducive to a physician spending the amount of time that may be required to provide individualized, patient-specific education and reassurance, this role matches the education and skill set of a physical therapist. [9] Physical therapists have been practicing internationally in EDs for over 20 years and have recently become more prevalent in EDs in the United States. [9,10] As ED patient volumes continue to increase, the number of patients who may potentially benefit from ED-based physical therapy (ED-PT) increased proportionally. [11]

Existing data suggest that ED-PT has a positive effect on a number of clinical and operational outcomes and is viewed positively by both patients and physicians. [12–14] In the primary care setting, early physical therapy (PT) appears to be associated with lower utilization of advanced imaging, lower rates of lumbar spinal injections and lumbar spinal surgery, and improved patient satisfaction. [15–19] In the ED, a primary contact physical therapy model has been shown to reduce wait times and improve patient flow, directing patients to early effective care, and freeing emergency physicians to focus on other emergent cases. [20–24] A primary contact model involves the Physical Therapist acting as primary provider and autonomous practitioner. This practice is now commonplace in the UK, Canada and Australia. It contrasts with the predominant model seen in US EDs in which the Physical Therapist acts as a ‘secondary contact practitioner’ after initial examination by an ED Physician and medical referral. [22,25] Furthermore, ED-PT has been suggested as an alternative to opioid prescribing for pain management in the ED. [26,27] In the primary care setting, early PT referral has been shown to offer reductions in longer-term opioid use and resulted in lower-intensity opioid use for patients with atraumatic lower back pain, although studies to date have failed to show benefit in the context of the ED. [28]

The purpose of this study was to determine the impact of ED-PT on overall rates of imaging, rates of opioid prescribing and length of stay when compared with patients not receiving PT. We hypothesize that patients who receive ED-PT would demonstrate reduced imaging rates, opioid prescribing and length of stay.

## Methods

### Study design and setting

The study design is that of a prospective observational study at the University of Utah ED over an 18-month period. We prospectively identified ED patients presenting with a musculoskeletal complaint to the University of Utah ED between January 2017 and July 2018. We reviewed the medical record to compare ED length of stay, imaging rates, and opioid administration among patients who received ED-PT to those who did not receive PT. The University of Utah Institutional Review Board approved the Study.

The University of Utah ED is an urban, academic ED located in Salt Lake City, Utah, with approximately 50,000 patient visits per year. At present, the University of Utah ED follows a traditional collaborative model of care, with ED providers consulting the physical therapist at their discretion. At the time of data collection there was one dedicated physical therapist working in the ED three days per week between the hours of 9 am and 6 pm. This individual was present through the entire study period.

### Selection of participants

The ED physical therapist maintained a record of all patients with musculoskeletal complaints for whom he was consulted and who received ED-PT throughout the 18-month study period. Patients with non-musculoskeletal complaints such as Vertigo were recorded separately by the Physical Therapist and not included within this study. The record included patient complaint, demographic information, and information on the ED intervention. Concurrent with this, on days in which the ED physical therapist was not present, trained research associates (RAs) identified ED patients presenting with a potential musculoskeletal complaint based on the triage chief complaint throughout the 18-month study period. The control group of non-PT patients was identified during the same standard ‘daytime’ hours as ED-PT patients. However, unlike the ED-PT group, our control group is not comprehensive, and represents a convenience sample of patients presenting with a musculoskeletal complaint. RAs approached these patients to clarify the nature of their complaint and to assure this was musculoskeletal in nature. RAs also confirmed with the primary patient provider that the complaint was indeed musculoskeletal in etiology. RAs recorded demographic and clinical information at the time of the ED visit. Any patient within this group who received ED-PT was excluded from the control group. Only patients with a final diagnosis of a primary musculoskeletal issue were included in the study.

### Methods and measurements

After the identification of appropriate patients, trained study personnel reviewed the electronic medical record and extracted specific pre-determined data points using a secure electronic spreadsheet. Data abstractors and analyzers were not blinded to patient group. These included medications administered in the ED, medications prescribed on discharge, total length of ED stay, and imaging performed in the ED. Medications and imaging studies were ordered by the primary ED provider only. The final ICD-10 code classification was also reviewed and confirmed to be musculoskeletal. Drugs were categorized according to the Multum Medisource Lexicom. For ease of classification, Tramadol is classified as a classic Opioid. Opioid combination medications such as hydrocodone-acetaminophen and oxycodone-acetaminophen are categorized as opioids only and are not counted towards the acetaminophen total.

### Outcomes

The primary study outcomes were total imaging rates (X-ray, CT and MRI), opioid administration in the ED, outpatient opioid prescription, and ED length of stay. We selected these as our primary outcomes given our hypothesis that ED initiated PT would reduce imaging, opioid use, and length of ED stay.

### Intervention

The University of Utah currently employs a ‘secondary contact practitioner model’. This is typical of almost all ED-PT programs within the US. All patients in the ED-PT group were seen by a single physical therapist after being initially assessed by a physician, nurse practitioner, or physician assistant. Providers at the University of Utah are encouraged to involve a physical therapist early in the presentation of a patient where PT is likely to be beneficial, such as in atraumatic lower back pain. Early assessment and intervention by a physical therapist, prior to any further intervention or work up, is our current model of practice, and may account for any differences seen between ED-PT and non-PT groups.

PT assessment included utilizing a motivational interviewing style to solicit a narrative history from the patient regarding their chief complaint as well as any factors they felt were contributory. Physical assessment included red flag screening and, when able, movement assessment for patterns that aggravated and eased symptoms. Patient management included education regarding the nature of musculoskeletal pain, reassurance of the lack of red flag findings, discussion of the natural course of musculoskeletal pain, advice on self-management strategies (including pacing and graded return to functional activity and activities of daily living as early as tolerated), avoidance of bed rest and therapeutic interventions based on the results of directional preference testing that most commonly involved gentle manual therapy in the form of mobilization to facilitate movement. Unless there was clear evidence of the cause of symptoms such as trauma, effort was made to avoid the use of biomedical or tissue-based explanations as the source of pain. Follow-up with outpatient PT was typically discussed and offered as an option if the patient or therapist felt it was appropriate or needed.

### Analysis

We performed univariate data analysis utilizing descriptive statistics with data presented as percentages for categorical variables and means for continuous variables. We evaluated differences between groups of categorical variables utilizing the Pearson's chi-square test and differences between continuous variables using Student's t-test. We present results using odds ratios (ORs) and 95% confidence intervals (CIs), with a p-value <0.05 considered statistically significant.

In order to account for potential differences between groups of patients, we performed a subgroup analysis on the subset of patients presenting with atraumatic low back pain and matched patients by age and gender. Given the larger number of non-PT patients in our study, we matched all non-PT patients with low back pain with PT patients with low back pain through matching by gender then matching by age +/- 1 year. We compared ED length of stay, imaging rates, and opioid administration rates between those who had ED-PT and those who did not. We performed analysis using STATA version 12.0 (StataCorp, College Station, TX) and VassarStats Website for Statistical Computation (vassarstats.net). The manuscript adheres to Strengthening the Reporting of Observational Study (STROBE) guidelines for the presentation of observational studies. [29]

### Ethics statement

This study was approved by the University of Utah Institutional Review Board. Informed consent was waived by the Institutional Review Board Committee. All patient records were anonymized prior to access.

## Results

Over the 18-month period, we identified 524 patients who presented to the ED with musculoskeletal pain and were included in the study. A total of 381 patients (72.7%) received ED-PT, whereas 143 patients (27.3%) did not receive PT. The PT and non-PT groups were similar in average age (42.8 years vs. 45.1 years, p = 0.155), gender (percent female: 53% vs. 46.9%, p-0.209), race and average presenting pain score. [Table 1] Primary presenting chief complaint was also similar between groups (cervical, thoracic, or lumbar pain: 57.7% vs. 53.1%, p = 0.345). [Table 2]

**Table 1:** Baseline characteristics of study population.

	Physical Therapy (n = 381)	No Physical Therapy (n = 143)	p value
Age, years	42.8	45.1	0.155
Female Sex	202 (53%)	67 (46.9%)	0.209
Race			
White	330 (86.6%)	125 (87.4%)	0.920
Hispanic	45 (11.8%)	18 (12.6%)	
Other	6 (1.6%)	0	
Insurance Type			
Commercial	267 (70%)	93 (65%)	0.314
Medicare	33 (8.7%)	14 (9.8%)	0.689
Medicaid	50 (13.1%)	18 (12.6%)	0.862
Self-Pay	31 (8.2%)	18 (12.6%)	0.119
Initial Pain Score, 0–10 scale	7.0	7.1	0.552

**Table 2:** Comparison of presenting chief complaint.

Complaint	Physical therapy performed in ED	No physical therapy in ED	p-value
Cervical, thoracic, or lumbar pain	57.7%	53.1%	0.345
Lower extremity pain	13.1%	24.5%	0.001
Upper extremity pain	12.1%	8.4%	0.232
Chest wall pain	2.1%	2.1%	0.649
Other complaint	14.9%	11.9%	0.368

**Fig 1:**
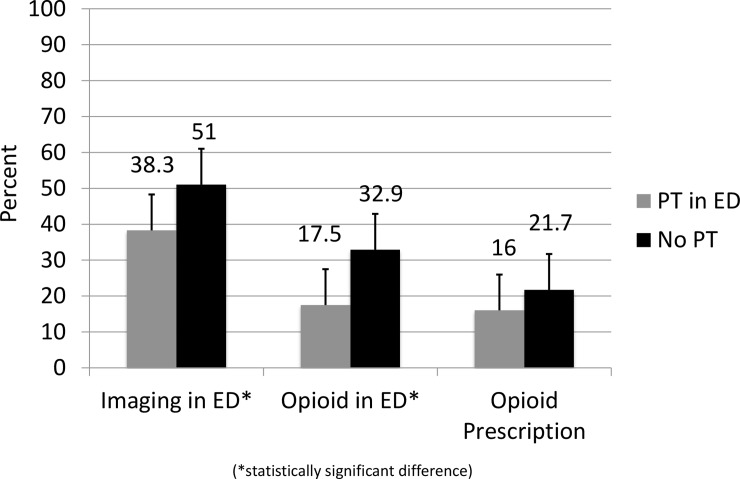
Imaging and opioid administration rates.

Average ED length of stay was significantly lower in the PT group when compared to the non-PT group (4 hours vs. 6.2 hours, p<0.001, range 1–26 hours vs. 1–28 hours). [Overall rates of imaging were also significantly lower in the PT group (38.3% vs. 51% p = 0.009). PT patients were less likely to have an x-ray performed in the ED (30.0% vs 43.4%, p = 0.00452). However, the rates of CT scanning (8.1% vs 11.2%, p = 0.27572) and MRI (4.7% vs 4.2%, p = 0.79486) did not differ significantly between the two groups. [Fig 1]

PT patients were less likely to receive and opioid pain medication in the ED (17.5% vs. 32.9%, p<0.001). PT patients were also less likely to receive acetaminophen in the ED (5.8% vs 11.9%, p = 0.01732) but had comparable rates of NSAID administration (21.5% vs 28.7%, p = 0.08544). Rates of outpatient opioid prescriptions were similar between the two groups (16% vs. 21.7%, p = 0.129). [Fig 1]

ED-PT patients received fewer benzodiazepines in the ED (92% vs 16.8%, p = 0.0217); however, benzodiazepine prescribing at the point of discharge was not significantly different (3.9% vs 4.2%, p = 0.9203). Topical Lidocaine preparations were infrequently prescribed in both ED-PT and non-PT groups both in the ED (0.3% vs 0%, p = 0.6033) and at discharge (0.3% vs 2.1%, p = 0.1124). Gabapentin was prescribed to fewer patients in the ED-PT group at discharge (1.8% vs 7.7%, p = 0.0023).

**Fig 2:**
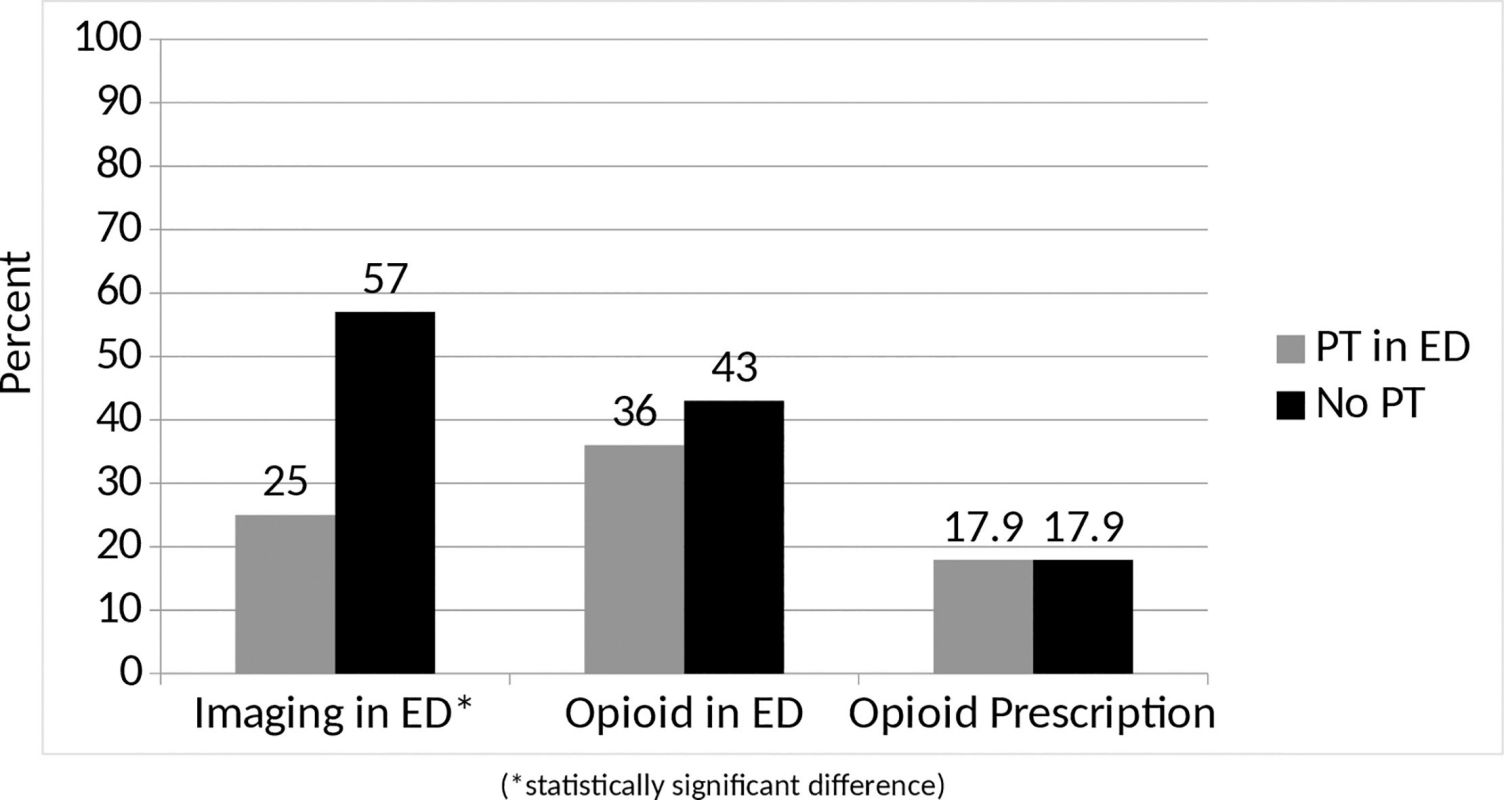
Subgroup comparison of matched patients with low back pain.

We performed a subgroup analysis of patients presenting with low back pain and matched patients by age and gender. We identified 112 patients with atraumatic low back pain for this matched comparison (PT 56 vs. non-PT 56). Average age in each group was 42 years (range PT 18–82 vs. non-PT 18–81) and 50% of each group was female. Average ED length of stay was similar between groups (PT 3.7 hours vs. non-PT 4.6 hours, p = 0.21). ED-PT patients received fewer imaging studies (PT 25% vs. non-PT 57%, p = 0.029) but had similar rates of opioid administration in the ED (PT 36% vs. non-PT 43%, P = 0.792) and outpatient opioid prescriptions (17.9%. vs 17.9%, p = 1.0). [Fig 2]

## Discussion

We found that patients who received ED-PT had a shorter ED length of stay, lower overall imaging rates, and fewer opioids administered in the ED. This study is the first to focus on the utilization of imaging at the discrete point of the ED visit, and it identifies an association between PT and decreased diagnostic imaging rates and therefore cost.

A number of large cohort and descriptive studies have demonstrated an association between the early initiation of PT in lower back pain and reduced downstream healthcare utilization. [18] Fritz et. al., using data extracted from a large database of employee sponsored health plans, retrospectively analyzed the effects of early (< 14 days) versus delayed (>14 days) PT on downstream healthcare utilization in patients with a first presentation of low back pain (LBP) to primary care providers. LBP-related healthcare costs were analyzed over an 18-month follow up period. Early physical therapy timing was associated with decreased risk of advanced imaging (odds ratio [OR] = 0.34, 95% confidence interval [CI]: 0.29, 0.41), additional physician visits (OR = 0.26, 95% CI: 0.21, 0.32), surgery (OR = 0.45, 95% CI: 0.32, 0.64), injections (OR = 0.42, 95% CI: 0.32, 0.64), and opioid medications (OR = 0.78, 95% CI: 0.66, 0.93) compared with delayed physical therapy. [19] This study is more recently supported by the work of Liu et. al. who demonstrated, in a retrospective cohort study of 6668 patients with LBP receiving PT in a community primary care setting, an association between early PT initiation (< 3 days) and downstream healthcare utilization costs. [30]

Of note, in our subgroup analysis of patients presenting with low back pain, the differences in opioid administration rates and ED length of stay were not present. The difference in imaging rates was more pronounced in this subgroup of patients than in the overall population, which may speak to the potential impact of PT consultation on imaging rates among patients with low back pain. It is worth noting that PT patients did have shorter ED length of stay and lower opioid administration rates during the ED stay, but these differences were not statistically significant. This may be due to the smaller size of the groups, and the analysis may have been underpowered to detect these differences in this subset of patients.

Initiating PT in the ED provides patients with meaningful intervention at the earliest possible stage of their presentation. [16,31] The ED often represents the first point of contact with the healthcare system for a given patient. Seeing a physical therapist in the ED offers a unique opportunity to provide guideline-adherent care for musculoskeletal pain from the outset, with the potential to realize the benefits of education, early mobility, and reduced exposure to unnecessary imaging. Some patients who may benefit from seeing a physical therapist may not otherwise have access to PT, potentially due to regulatory and health insurance restrictions or simply due to patient awareness of this resource. [18] Having PT available in ED may mitigate some of these barriers and increase the likelihood that patients have the opportunity to receive this care.

ED-PT as part of a primary contact provider model has not been shown to increase ED length of stay. [12,32] However, there remains concern that secondary contact provider models, such as that in our institution, may prolong lengths of stay. [13,33] In our study, ED-PT was associated with shorter ED length of stay. The reason for this finding is likely multifactorial; fewer imaging studies in the PT group and our institutional emphasis on early PT consultation were likely the greatest contributors. A significant contributor to length of stay in some ED programs is likely delayed referral to PT. Providers in our institution are encouraged to refer to PT early and avoid using ED-PT as a ‘last resort’ after exhausting alternative measures (opioid analgesia, diagnostic imaging). As EDs see increasing volumes of patients, alternative and creative ways to reduce wait times and improve patient flow become increasingly important. [32] Our study demonstrates that early referral and PT involvement may provide a strategy to achieve this goal in specific patient populations.

Finally, identifying treatments and therapies that reduce the need for opioid prescribing is invaluable in the midst of the current opioid epidemic. [26,27,34] Back pain is the most frequent diagnosis for which opioids and benzodiazepines are prescribed in the ED. [34] PT has been suggested as an alternative management strategy for managing both acute and chronic pain. [17] A recent study by Kim et. al. was the first to focus on the effect of ED-PT on opioid prescribing in back and neck pain in the ED setting. [28] They found that individuals presenting to the ED with back and neck pain who were seen by a physical therapist during the visit were just as likely to receive an opioid prescription. Similarly, we found no significant difference in outpatient opioid prescribing but did note a substantial difference in opioid administration while patients were in the ED. In our ED PT program, we have encouraged physicians to consult PT prior to initiating pharmaceutical therapy, which may be a factor in the differing opioid administration levels within the ED itself. However, the present study and that of Kim et al demonstrate no association between ED-PT and reduced opioid prescribing at the point of discharge. This contrasts with a number of claims-based studies indicating lower opioid prescribing in those patients engaging in PT. [35,36] However, these studies focus on outpatients, in whom our population of patients in the ED may significantly differ, both in terms of urgency of encounter and acute pain score. It may also be the case that PT has a more protective effect against opioid prescribing long-term, and is less effective as an opioid substitution therapy acutely at the point of ED discharge Further research may clarify the comparable rates of outpatient opioid prescribing between those who receive ED-PT and those who do not.

## Limitations

Our study is limited to a single center, and all patients were seen by a single physical therapist. As such, our findings may be unique to this center, to the musculoskeletal diagnostic evaluations, and to the management strategies at this site.

Our methodology to identify study patients meant that the scope of the PT patients included in the study was comprehensive and represented all patients for whom ED-PT was provided during the 18-month study period. However, our control group represented a convenience sample of patients presenting with a musculoskeletal complaint. Certain limitations may be inherent to the methodology employed to identify appropriate patients for comparison. Given that our physical therapist did not assess the non-PT group, it is possible that our non-PT group is fundamentally different from the group that received PT, and that the non-PT group contains a sub-set of patients who would not have been amenable to ED initiated PT and for whom the physical therapist would not have been consulted if available. Given the limited presence of the physical therapist in the ED, we felt that this offered an appropriate arrangement to recruit a group of similar patients who presented to the ED on days in which the physical therapist was not present and, as such, would form an appropriate control group. Still, this methodology does not utilize a robust randomization process in assigning patient to PT and may have selected for a more complex or unique group of patients among those who did not have PT in the ED.

Additional limitations relate to the reliance upon the electronic medical record for patient demographic, testing, and medication information. While we identified patients prospectively either through the research associates or through the ED physical therapist, we retrieved relevant outcomes through review of the medical record. As such, this methodology is susceptible to bias through mistakes within the medical record or error in the review and transcription process.

This study focuses on overall rates of imaging and does not assess the appropriateness or inappropriateness of the imaging studies performed. We know that many patients with musculoskeletal complaints receive unnecessary imaging investigations, however only overall rates of imaging are compared in this study, and therefore no conclusions can be made about whether PT reduces unnecessary imaging.

In focusing solely on outcomes at the time of the ED encounter, our study is further limited by the lack of ongoing, longitudinal patient follow-up after the ED visit. It is unclear whether the benefits of PT seen in the ED translate into ongoing benefits to the patient or the community, such as reduced use of healthcare resources and the ongoing reduced use of opioids. Such longitudinal endpoints may form the basis for future inquiry.

## Conclusion

Our study found that patients who had ED-PT had a shorter length of stay, less overall imaging, and reduced rates of ED opioid administration. However, we found no significant difference in outpatient opioid prescriptions. This study is the first ED-based study to demonstrate an impact of ED-PT on opioid administration and may provide future direction for research assessing ED opioid reduction strategies. Similarly, the impact on overall healthcare utilization through imaging and length of stay suggests added benefits of ED-PT beyond the therapeutic relationship established and care provided within the ED setting.

## References

[1] James SL, Abate D, Abate KH, Abay SM, Abbafati C, Abbasi N, et al Global, regional, and national incidence, prevalence, and years lived with disability for 354 diseases and injuries for 195 countries and territories, 1990–2017: a systematic analysis for the Global Burden of Disease Study 2017. The Lancet. 2018 11;392(10159):1789–858. 10.1016/S0140-6736(18)32279-7 PMC6227754 30496104

[2] Edwards J, Hayden J, Asbridge M, Gregoire B, Magee K. Prevalence of low back pain in emergency settings: a systematic review and meta-analysis. BMC Musculoskelet Disord. 2017 12;18(1):143 10.1186/s12891-017-1511-7 28376873 PMC5379602

[3] McCaig LF, Nawar EW. National Hospital Ambulatory Medical Care Survey: 2004 emergency department summary. Adv Data. 2006 6 23;(372):1–29. 16841785

[4] CDC. National Hospital Ambulatory Medical Care Survey [Internet]. 2011. Available from: https://www.cdc.gov/nchs/data/ahcd/nhamcs_emergency/2011_ed_web_tables.pdf

[5] Chou R, Qaseem A, Snow V, Casey D, Cross JT, Shekelle P, et al Diagnosis and treatment of low back pain: a joint clinical practice guideline from the American College of Physicians and the American Pain Society. Ann Intern Med. 2007 10 2;147(7):478–91. 10.7326/0003-4819-147-7-200710020-00006 17909209

[6] Qaseem A, Wilt TJ, McLean RM, Forciea MA, for the Clinical Guidelines Committee of the American College of Physicians. Noninvasive Treatments for Acute, Subacute, and Chronic Low Back Pain: A Clinical Practice Guideline From the American College of Physicians. Ann Intern Med. 2017 4 4;166(7):514 10.7326/M16-2367 28192789

[7] Darlow B. Beliefs about back pain: The confluence of client, clinician and community. International Journal of Osteopathic Medicine. 2016 6;20:53–61.

[8] O’Connell NE, Cook CE, Wand BM, Ward SP. Clinical guidelines for low back pain: A critical review of consensus and inconsistencies across three major guidelines. Best Practice & Research Clinical Rheumatology. 2016 12;30(6):968–80. 29103554 10.1016/j.berh.2017.05.001

[9] Lebec MT, Jogodka CE. The Physical Therapist as a Musculoskeletal Specialist in the Emergency Department. J Orthop Sports Phys Ther. 2009 3;39(3):221–9. 10.2519/jospt.2009.2857 19252261

[10] Guy R, Lebec M. Characteristics of known emergency department physical therapist consultation programs: A cross-sectional descriptive study to provide administrative guidance. PT Journal of Policy, Administration and Leadership. 2019;19(2).

[11] Lebec MT. Hospital employs emergency department-based physical therapists, leading to improved quality, efficiency, and patient/physician satisfaction. Service delivery innovation profile [Internet]. Agency for Healthcare Research and Quality; 2010 Available from: https://innovations.ahrq.gov/profiles/hospital-employs-emergency-department-based-physical-therapists-leading-improved-quality

[12] McClellan CM. Effect of an extended scope physiotherapy service on patient satisfaction and the outcome of soft tissue injuries in an adult emergency department. Emergency Medicine Journal. 2006 5 1;23(5):384–7. 10.1136/emj.2005.029231 16627842 PMC2564090

[13] Michael T Lebec Steven Cernohous, Tenbarge Lisa, Gest Colleen, Severson Kristen, Howard Sharon. Emergency Department Physical Therapist Service: A Pilot Study Examining Physician Perceptions. The Internet Journal of Allied Health Sciences and Practice. 2010 1;8(1).

[14] Kilner E, Sheppard L. The ‘lone ranger’: a descriptive study of physiotherapy practice in Australian emergency departments. Physiotherapy. 2010 9;96(3):248–56. 10.1016/j.physio.2010.01.002 20674658

[15] Sun E, Moshfegh J, Rishel CA, Cook CE, Goode AP, George SZ. Association of Early Physical Therapy With Long-term Opioid Use Among Opioid-Naive Patients With Musculoskeletal Pain. JAMA Netw Open. 2018 12 14;1(8):e185909 10.1001/jamanetworkopen.2018.5909 30646297 PMC6324326

[16] Childs JD, Fritz JM, Wu SS, Flynn TW, Wainner RS, Robertson EK, et al Implications of early and guideline adherent physical therapy for low back pain on utilization and costs. BMC Health Serv Res. 2015 6;15(1):150. 25880898 10.1186/s12913-015-0830-3 PMC4393575

[17] Sohil P. Potential impact of early physiotherapy in the emergency department for non-traumatic neck and back pain. World J Emerg Med. 2017;8(2):110 10.5847/wjem.j.1920-8642.2017.02.005 28458754 PMC5409230

[18] Frogner BK, Harwood K, Andrilla CHA, Schwartz M, Pines JM. Physical Therapy as the First Point of Care to Treat Low Back Pain: An Instrumental Variables Approach to Estimate Impact on Opioid Prescription, Health Care Utilization, and Costs. Health Serv Res. 2018 12;53(6):4629–46. 10.1111/1475-6773.12984 29790166 PMC6232429

[19] Fritz JM, Childs JD, Wainner RS, Flynn TW. Primary Care Referral of Patients With Low Back Pain to Physical Therapy: Impact on Future Health Care Utilization and Costs. Spine. 2012 12;37(25):2114–21. 10.1097/BRS.0b013e31825d32f5 22614792

[20] Bird S, Thompson C, Williams KE. Primary contact physiotherapy services reduce waiting and treatment times for patients presenting with musculoskeletal conditions in Australian emergency departments: an observational study. Journal of Physiotherapy. 2016 10;62(4):209–14. 10.1016/j.jphys.2016.08.005 27637771

[21] Taylor NF, Norman E, Roddy L, Tang C, Pagram A, Hearn K. Primary contact physiotherapy in emergency departments can reduce length of stay for patients with peripheral musculoskeletal injuries compared with secondary contact physiotherapy: a prospective non-randomised controlled trial. Physiotherapy. 2011 6;97(2):107–14. 10.1016/j.physio.2010.08.011 21497244

[22] de Gruchy A, Granger C, Gorelik A. Physical Therapists as Primary Practitioners in the Emergency Department: Six-Month Prospective Practice Analysis. Physical Therapy. 2015 9 1;95(9):1207–16. 10.2522/ptj.20130552 25929528

[23] Ferreira GE, Traeger AC, Maher CG. Review article: A scoping review of physiotherapists in the adult emergency department: PHYSIOTHERAPISTS IN THE ADULT EMERGENCY DEPARTMENT. Emergency Medicine Australasia. 2019 2;31(1):43–57. 10.1111/1742-6723.12987 29664184

[24] Sutton M, Govier A, Prince S, Morphett M. Primary-contact physiotherapists manage a minor trauma caseload in the emergency department without misdiagnoses or adverse events: an observational study. Journal of Physiotherapy. 2015 4;61(2):77–80. 10.1016/j.jphys.2015.02.012 25801363

[25] Kersten P, McPherson K, Lattimer V, George S, Breton A, Ellis B. Physiotherapy extended scope of practice–who is doing what and why? Physiotherapy. 2007 12;93(4):235–42.

[26] Lynch MJ, Yealy DM. Looking Ahead: The Role of Emergency Physicians in the Opioid Epidemic. Annals of Emergency Medicine. 2018 6;71(6):676–8. 10.1016/j.annemergmed.2018.01.051 29459055

[27] Poon SJ, Greenwood-Ericksen MB. The Opioid Prescription Epidemic and the Role of Emergency Medicine. Annals of Emergency Medicine. 2014 11;64(5):490–5. 10.1016/j.annemergmed.2014.06.016 25017821

[28] Kim HS, Kaplan SH, McCarthy DM, Pinto D, Strickland KJ, Courtney DM, et al A comparison of analgesic prescribing among ED back and neck pain visits receiving physical therapy versus usual care. The American Journal of Emergency Medicine. 2018 10;S0735675718308258. 10.1016/j.ajem.2018.10.009 30528050

[29] Von Elm E, Altman DG, Egger M, Pocock SJ, Gøtzsche PC, Vandenbroucke JP, et al The Strengthening the Reporting of Observational Studies in Epidemiology (STROBE) statement: guidelines for reporting observational studies. Ann Intern Med. 2007 10 16;147(8):573–7. 10.7326/0003-4819-147-8-200710160-00010 17938396

[30] Liu X, Hanney WJ, Masaracchio M, Kolber MJ, Zhao M, Spaulding AC, et al Immediate Physical Therapy Initiation in Patients With Acute Low Back Pain Is Associated With a Reduction in Downstream Health Care Utilization and Costs. Physical Therapy. 2018 5 1;98(5):336–47. 10.1093/ptj/pzy023 29669083

[31] Alexandria VA. Incorporating physical therapist practice in the ED: a toolkit for practitioners [Internet]. American Physical Therapy Association; 2011 Available from: https://www.apta.org/EmergencyDepartment/

[32] Fleming-McDonnell D, Czuppon S, Deusinger SS, Deusinger RH. Physical Therapy in the Emergency Department: Development of a Novel Practice Venue. Physical Therapy. 2010 3 1;90(3):420–6. 10.2522/ptj.20080268 20056722

[33] Fruth SJ, Wiley S. Physician Impressions of Physical Therapist Practice in the Emergency Department: Descriptive, Comparative Analysis Over Time. Physical Therapy. 2016 9 1;96(9):1333–41. 10.2522/ptj.20150306 27055541

[34] Ballantyne JC. Avoiding Opioid Analgesics for Treatment of Chronic Low Back Pain. JAMA. 2016 6 14;315(22):2459 10.1001/jama.2016.6753 27299620

[35] Thackeray A, Hess R, Dorius J, Brodke D, Fritz J. Relationship of Opioid Prescriptions to Physical Therapy Referral and Participation for Medicaid Patients with New-Onset Low Back Pain. J Am Board Fam Med. 2017 11;30(6):784–94. 10.3122/jabfm.2017.06.170064 29180553

[36] Zheng P, Kao M-C, Karayannis NV, Smuck M. Stagnant Physical Therapy Referral Rates Alongside Rising Opioid Prescription Rates in Patients With Low Back Pain in the United States 1997–2010: SPINE. 2017 5;42(9):670–4. 10.1097/BRS.0000000000001875 28441685 PMC9853341

